# The Next Meeting of the American Medical Association

**Published:** 1902-10

**Authors:** 


### THE NEXT MEETING OF THE AMERICAN MEDICAL ASSOCIATION.

THE next meeting of the American Medical Association will be held in New Orleans May 5 to 8, 1903.

We publish in another place a letter from Dr. Isadore Dyer, chairman of the Committee of Arrangements.

New Orleans has a reputation for hospitality, and there are many extra-medical attractions in the city which render it very interesting. It is hoped that the meeting will be a notable one, and one which the profession in the South can remember with pride.

---

THE Elkin-Watson Drug Co. have a placard conspicuously posted in their store which reads: "We do not believe, nor do we indulge, in counter prescribing. If you are sick and don't know what you want, call in a physician."

This is a step in the right direction, and an example that should be emulated by every honest druggist in the country.

---

THE marriage of Dr. Joseph Nisbet Le Conte and Miss Lillian King will take place at the home of the bride's parents in Inman Park, on October 8. Congratulations.

